# Measles Outbreak, the Netherlands, 2008

**DOI:** 10.3201/eid1602.090114

**Published:** 2010-03

**Authors:** Susan Hahné, Margreet J.M. te Wierik, Liesbeth Mollema, Eva van Velzen, Eric de Coster, Corien Swaan, Hester de Melker, Robert van Binnendijk

**Affiliations:** National Institute for Public Health and the Environment (RIVM), Bilthoven, the Netherlands (S. Hahné, L. Mollema, C. Swaan, H. de Melker, R. van Binnendijk); Municipal Health Service, The Hague, the Netherlands (E. van Velzen, E. de Coster ); Municipal Health Service, Utrecht, the Netherlands (M.J.M. te Wierik)

**Keywords:** Viruses, measles, the Netherlands, letter

**To the Editor**: From June 1 through October 16, 2008, an outbreak of 99 reported measles cases occurred in the Netherlands (*1*). This outbreak was the largest measles outbreak in the Netherlands since 1999–2000, when >3,200 cases, including 3 deaths, were reported (*2*).

In the Netherlands, clinical symptoms compatible with measles in a person with laboratory-confirmed measles virus infection or an epidemiologic link to a laboratory-confirmed case are notifiable (i.e., must be reported to public health authorities). The National Measles Reference Laboratory conducts genotyping and submits sequences to the World Health Organization European Region Measles Nucleotide Surveillance database (www.hpa-bioinformatics.org.uk/Measles/Public/Web_Front/main.php).

Of the 99 measles cases reported in the 2008 outbreak, 40 were laboratory confirmed and 59 were notified based on an epidemiologic link. The first case-patient in the outbreak was a 6-year-old unvaccinated resident of The Hague who had not been abroad in the month before onset of illness. The source of her infection was unknown. She attended a school based on anthroposophic principles; the school had an estimated measles-mumps-rubella (MMR) vaccination coverage of 80% (M. Monné-van Wirdum, pers. comm.). Subsequently, 52 additional cases were reported from this and from another anthroposophic school in The Hague (cluster 1; Figure A1). Two months after the first case, 22 additional cases were reported associated with an anthroposophic summer camp in the east of the Netherlands (cluster 2; Figure A1). Five additional cases had an epidemiologic link with an anthroposophic summer camp in France (cluster 3, 2 cases; Figure A1) and Switzerland (cluster 4, 3 cases; Figure A1). No known measles patients in Switzerland were linked to this cluster (J. Richard, pers. comm.). Subsequently, 12 cases were reported that were associated with 2 daycare centers in the city of Utrecht (cluster 5 and 6), both linked to an anthroposophic community. From all 6 clusters and from 2 of the 7 cases with an unknown source, indistinguishable measles viruses (genotype D8, 22 cases) were identified. Given the low prevalence of this strain in Europe (J. Kremer, pers. comm.), we concluded that virus transmission occurred between all 6 clusters. The first cluster was not epidemiologically linked to any of the recent outbreaks in anthroposophic groups in Europe (*3*).

No case had an epidemiologic link to more than 1 cluster, suggesting the 6 cases introducing measles into these clusters were unreported. When the 7 cases with an unknown source are considered, this finding suggests that at least 13 cases were not reported (maximum reporting completeness 88%). However, transmission through patients with subclinical cases may also have played a role (*4*).

There were no deaths. Four case-patients (4%) were admitted to hospitals. The median age was 9 years (range 8 months–48 years). Of the 98 case-patients with information on vaccination status, 91 (93%) had been unvaccinated, 6 (6%) had had 1 dose, none (0%) had had 2 doses, and 1 (1%) had had 3 doses before onset of illness. One of the 6 case-patients, vaccinated only once, had received her first MMR vaccine only 11 days before the date of onset of illness and is hence not considered a vaccine failure. Of all 99 case-patients, 91% had been eligible for >1 MMR vaccination according to the vaccination schedule in the Netherlands. Of these cases, available information for 84 case-patients indicated 48% (40 persons) were reported to be unvaccinated because of their anthroposophic beliefs, 49% (41 persons) because of a critical attitude towards vaccination, and 4% (3 persons) for other reasons.

Outbreak control plans in the Netherlands focus on protecting the population by adjusting the vaccination schedule during a nationwide outbreak (*5*). Studies are ongoing into knowledge and attitudes toward vaccination in communities with low vaccination coverage, aiming to identify opportunities to improve coverage.

The outbreak remained largely restricted to persons with philosophical objections to MMR vaccination, which suggests that there are sufficient levels of herd immunity in the general population. Remarkably, no cases were reported from the Dutch Orthodox Reformed Church community, despite the low vaccine coverage in this group. This finding suggests that orthodox reformed and anthroposophic population subgroups have little direct contact, consistent with previous observations (*6*).

Measles vaccination was introduced in the Netherlands in 1976. The single-dose regimen was in 1987 replaced by a 2-dose regimen of MMR vaccine; the first dose at 14 months and the second at 9 years. The vaccination coverage for >1 MMR dose has been >95% from birth cohort 1986 onward (*7*)*.* During 2002–2007, the incidence of measles notifications in the Netherlands was below the World Health Organization regional threshold for elimination (1/1 million population/year) (*8*). Nevertheless, this outbreak demonstrates the continued risk for measles transmission in the Netherlands. This suggests that indicators based merely on incidence and national vaccination coverage are of limited usefulness for certification of measles elimination. Data on measles seroprevalence and mixing patterns that will soon be available from the second national seroprevalence study will provide more insight into the dynamics of measles transmission in a population with pockets of low vaccination coverage. These data will also help assess progress toward measles elimination from the Netherlands.

## Figures and Tables

**Figure A1:**
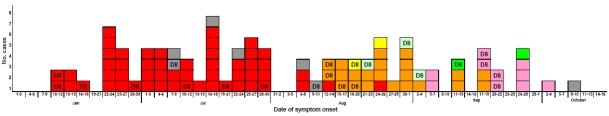
Cases of measles investigated, by date of onset of symptoms, cluster, and measles virus strain, the Netherlands, June 1–October 16, 2008 (n = 99). D8 indicates the outbreak strain (MVi/Den Haag.NLD/25.08, accession no. EU878303). Association of cases with attendance at schools, camps, and daycare centers: red, schools A and B, The Hague, the Netherlands; orange, anthroposophic summer camp C, Drenthe, the Netherlands; light green, anthroposophic summer camp D, Switzerland; yellow, anthroposophic summer camp E, France; pink, child day care center F, Utrecht, the Netherlands; dark green, child day care center G, Utrecht; gray, unknown.
